# Comparison among fertility-sparing therapies for well differentiated early-stage endometrial carcinoma and complex atypical hyperplasia

**DOI:** 10.18632/oncotarget.17588

**Published:** 2017-05-03

**Authors:** Qing Zhang, Gonghua Qi, Margaux J. Kanis, Ruifen Dong, Baoxia Cui, Xingsheng Yang, Beihua Kong

**Affiliations:** ^1^ Department of Obstetrics and Gynecology, Qilu Hospital, Shandong University, Ji’nan, Shandong, 250012, P.R. China; ^2^ Gynecology Oncology Key Laboratory, Qilu Hospital, Shandong University, Ji’nan, Shandong, 250012, P.R. China; ^3^ Division of Gynecologic Oncology, Department of Obstetrics and Gynecology, Northwestern University Feinberg School of Medicine, Chicago, IL, 60611, USA; ^4^ School of Medicine, Shandong University, Ji’nan, Shandong, 250012, P.R. China

**Keywords:** endometrial carcinoma, complex atypical hyperplasia, fertility-sparing therapy, hysteroscopic resection, oral progestogens

## Abstract

**Objective:**

To compare fertility-sparing therapies including oral progestogens, hysteroscopic resection (HR), and the levonorgestrel- releasing intrauterine system (LNG-IUS) in achieving disease regression, recurrence and live birth rate in well differentiate early-stage endometrial carcinoma (eEC) and complex atypical hyperplasia(CAH).

**Study Design:**

This was a meta-analysis of previous studies focus on the fertility-sparing therapy for well differentiate early-stage endometrial carcinoma (eEC) and complex atypical hyperplasia (CAH).

**Date Sources:**

Medline, the Cochrane Library and Embase was searched with the terms and Synonyms: words similar to eEC and CAH with therapies associated with fertility-sparing.

**Main Outcome Measures:**

The number of all patients accepted fertility sparing therapies, patients got regressed, relapsed and delivered were extracted from each study, and the regression, recurrence, and live birth rate of each study were calculated. The regression, recurrence and live birth rates between each two interventions were compared with the aid of meta-regression in packages of “meta” and ”meta for” written in R.

**Results:**

Fifty-four studies reported fertility sparing therapies in young women with eEC and CAH were included. Meta-analysis showed that HR followed by progestogens achieved a higher pooled regression (98.06% vs 77.20% *P* < 0.0001) and live birth rate (52.57% vs 33.38%, *P* = 0.0944) and a lower recurrence rate compared with oral progestogens alone (4.79% vs 32.17% *P* = 0.0004). At the same time, the pooled live birth rate (52.57% vs 18.09% *P* =0.0399) of HR followed by progestogens are significantly higher than the LNG-IUS alone. Which no statistical difference in regression (98.06% vs 94.24%; *P* = 0.4098) and recurrence rates (4.79% vs 3.90% *P* = 0.8561) was seen.

**Conclusions:**

Of the available fertility-sparing therapeutic options, HR followed by progestogens may be a more effective one.

## INTRODUCTION

Endometrial carcinoma (EC), the most common malignancy of the female genital tract, and complex atypical hyperplasia (CAH), its precursor lesion, most frequently affects peri- and post-menopausal women. It is reported that the incidence of EC among women 40 years or younger comprised 14.4% of all patients diagnosed with EC between 1976–1983 [1]. However, the incidence of young women who are diagnosed with EC and CAH is increasing, particularly as the rate of obesity increases and popularity of postpone delivery age. This poses a dilemma for those who wish to retain fertility as a total hysterectomy and bilateral salpingo-oophorectomy (TH/BSO) is the standard treatment. Recent studies have focused on fertility-sparing therapy for early-stage endometrial carcinoma (eEC) and CAH. Oral progestogens such as megestrol acetate (MA) and medroxyprogesterone acetate (MPA) were most frequently used [2], but in recent years, the levonorgestrel-releasing intrauterine system (LNG-IUS) [3], and hysteroscopic resection(HR) of the cancer or hyperplastic area followed by oral or intrauterine progestogens have been demonstrated to be safe and effective alternatives [4, 5]. However, previous reports of these are limited to small sample studies and meta-analysis [6, 7], and few focus on the comparison among these therapies [8, 9]. In this report, we systematically review previously published observational studies, relative reviews and meta-analyses to perform a meta-analysis in comparing among their treatment effects.

## RESULTS

**Figure 1:**
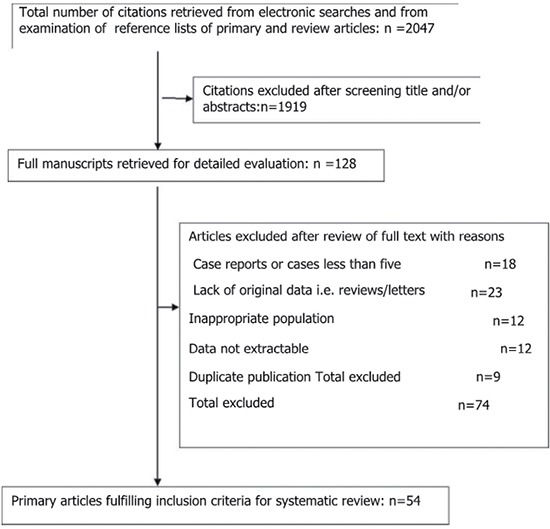
Study selection process

**Figure 2:**
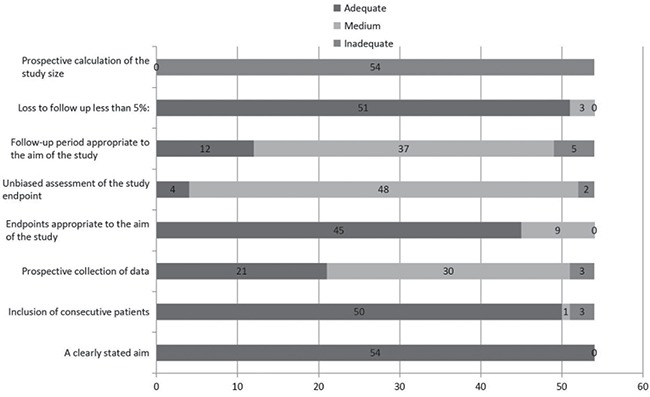
Quality assessment of the studies

The electronic search identified 2047 citations in Medline, the Cochrane Library and Embase. Of these, 1919 were excluded as the title and abstracts not meet the inclusion criteria. One hundred and twenty-eight publications were obtained, and another 74 were excluded for not meeting the inclusion criteria after thorough reading of the paper. In the final analysis, 54 articles [6, 10–62] were included. The process of article selection can be seen in Figure 1. Detailed information of the included studies and quality analysis are presented in See Supplementary Table 4 and Figure 2.

### Hysteroscopic resection with or without progestogens compare to oral progestogens alone

**Figure 3:**
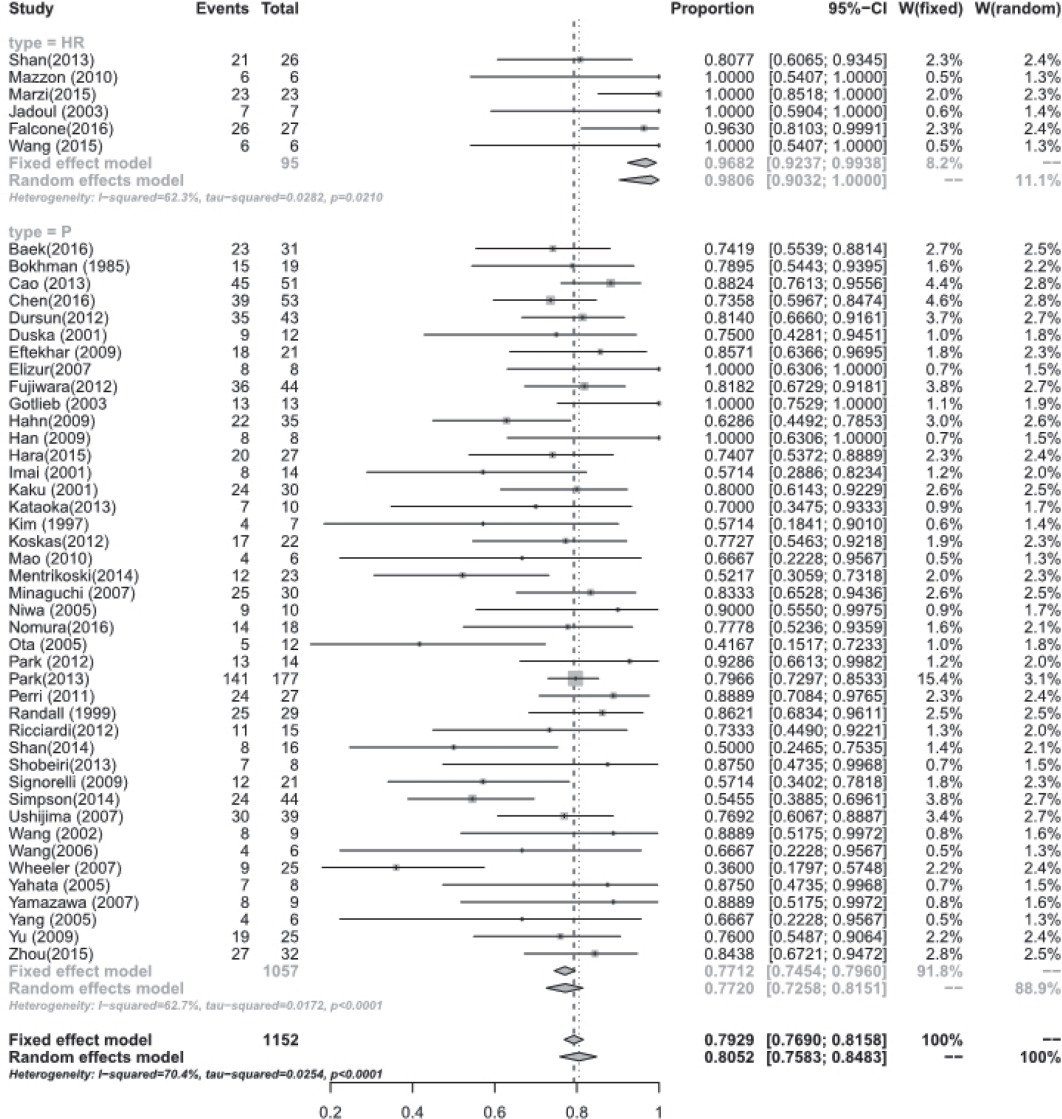
Regression rate between hysterscopic resection (HR) and oral progestogens (P)

**Figure 4:**
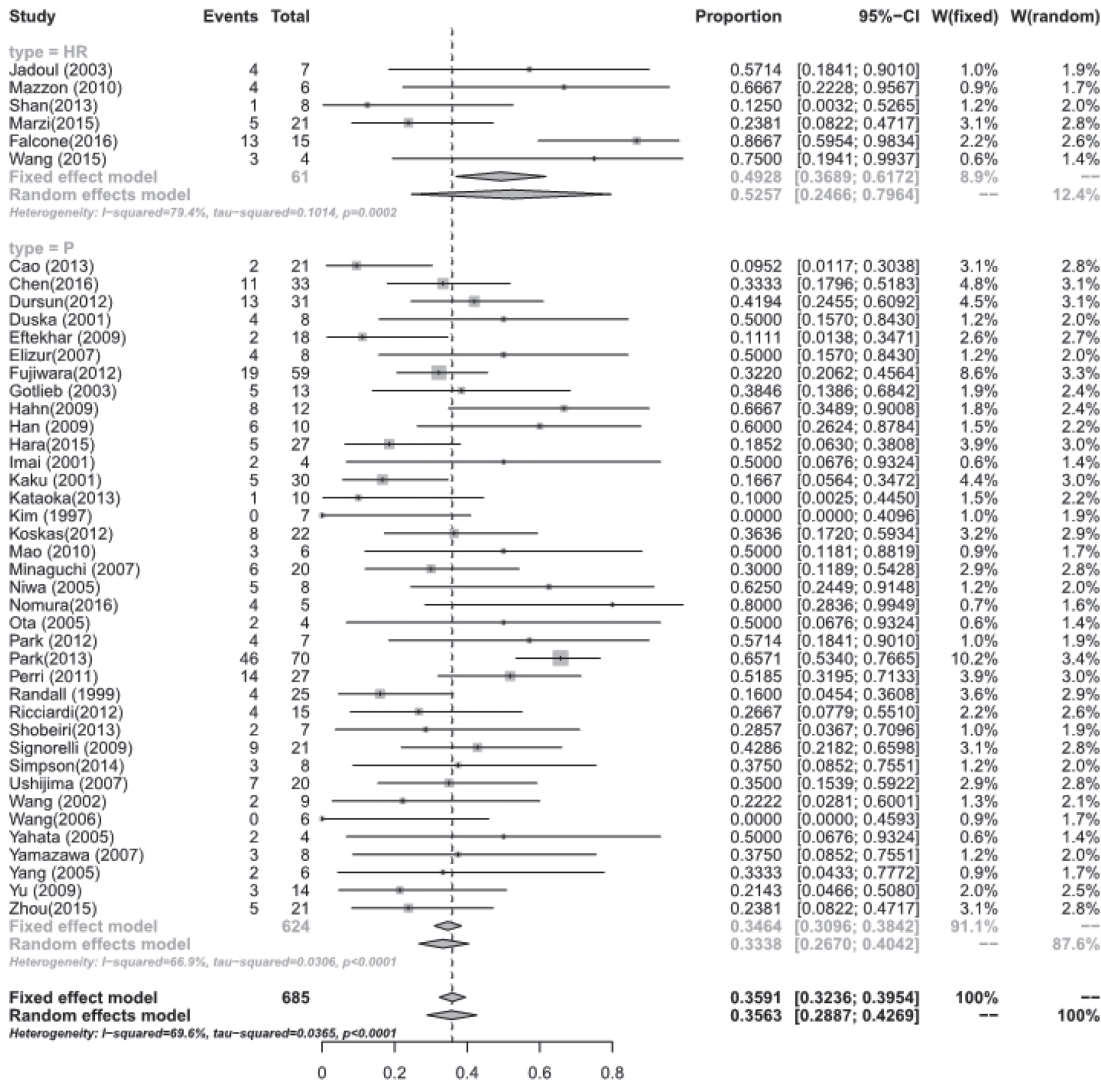
Live birth rate between hysterscopic resection (HR) and oral progestogens (P)

**Figure 5:**
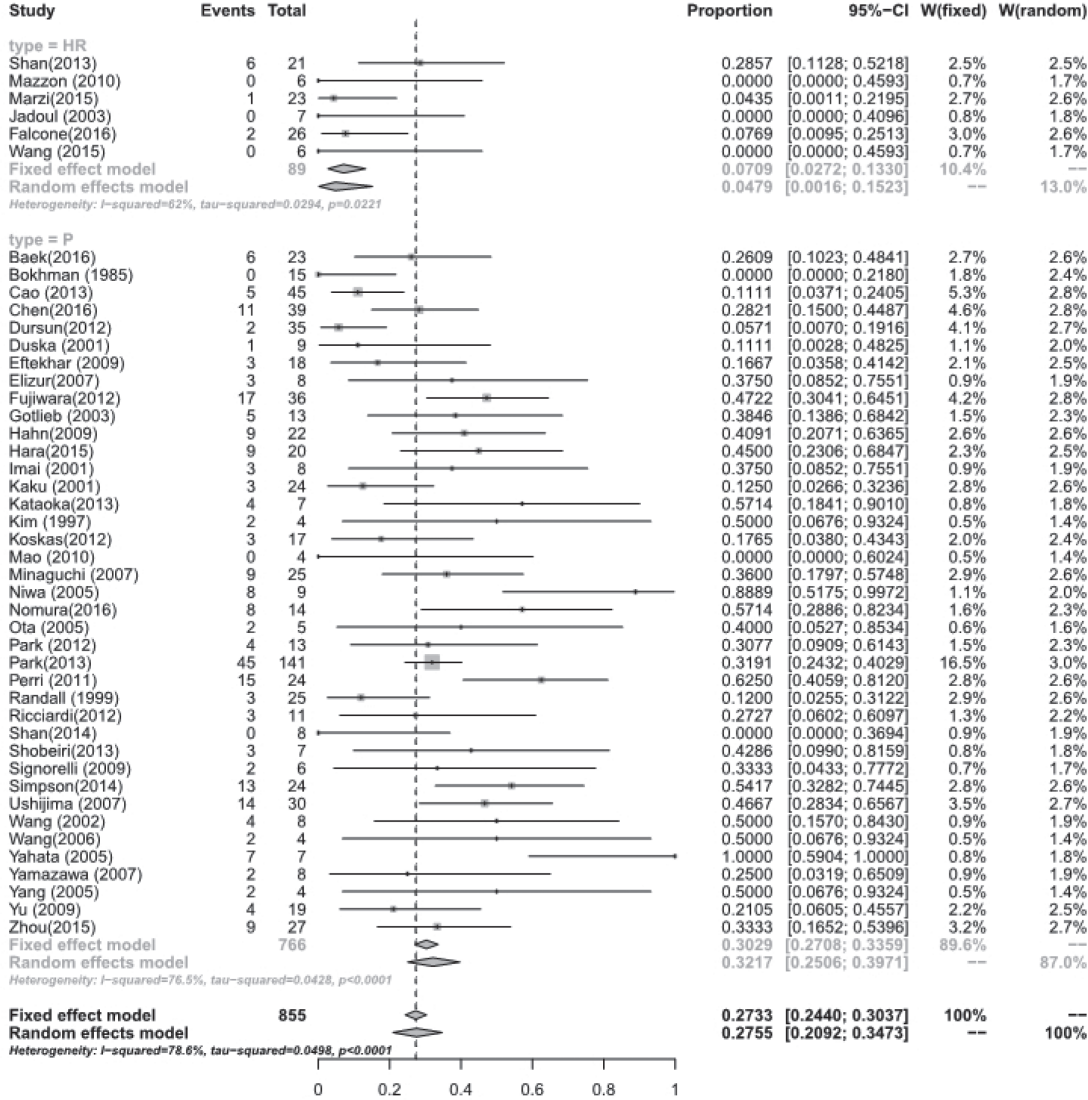
Recurrence rate between hysterscopic resection (HR) and oral progestogens (P)

Meta-analysis of the studies focusing on women with eEC and CAH treated with hysteroscopic resection(HR) with or without another methods such as progestogens or GnRH agonist showed a pooled regression, recurrence and live birth rate of 98.06% (95% [CI], 90.32–100.00), 4.79% (95% [CI], 0.16–15.23), and 52.57% (95% [CI], 24.66–79.64), respectively. Studies focusing on oral progestogens alone found a pooled regression, recurrence and live birth rate of 77.20% (95% [CI], 72.58–81.51), 32.17% (95% [CI], 25.06–39.71), and 33.38 % (95% [CI], 26.70–40.42), respectively. Meta-analysis showed that HR with or without progestogens achieved a statistical significantly higher pooled regression rate (*P* < 0.0001) (See Figure 3), and a slightly higher live birth rate (*P* = 0.0944) though without statistical sense (See Figure 4) and a statistically significant lower recurrence rate (*P* = 0.0004) (See Figure 5) compared with oral progestogens alone (See Table 1).

**Table 1:** Regression, recurrence and live birth rate of hysteroscopic resection (HR) and oral progestogens (p)

	Number of studies	Test for Heterogeneity (I^2^)	Test for Heterogeneity: (tau-squared)	Fixed effect model (effect size, 95% CI)	Random effects model (effect size, 95% CI)	*P* value
Regression rate						**< 0.0001**
In HR	6	62.3%	0.0210	0.9682 [0.9237; 0.9938]	0.9806 [0.9032; 1.0000]	
In P	45	62.7%	< 0.0001	0.7712 [0.7454; 0.7960]	0.7720 [0.7258; 0.8151]	
Recurrence rate						**0.0004**
In HR	6	62%	0.0221	0.0709 [0.0272; 0.1330]	0.0479 [0.0016; 0.1523]	
In P	39	76.5%	< 0.0001	0.3029 [0.2708; 0.3359]	0.3217 [0.2506; 0.3971]	
Live birth rate						**0.0944**
In HR	6	79.4%	0.0002	0.4928 [0.3689; 0.6172]	0.5257 [0.2466; 0.7964]	
In P	37	66.9%	< 0.0001	0.3464 [0.3096; 0.3842]	0.3338 [0.2670; 0.4042]	

### Hysteroscopic resection with or without progestogens and LNG- IUS alone

Meta-analysis of those with eEC and CAH treated with LNG-IUS have a pooled regression, recurrence and live birth rate of 94.24% (95% [CI], 83.23–99.60), 3.90% (95% [CI], 0.08–12.98) and 18.09% (95% [CI], 7.42–32.14), respectively. The pooled live birth rate (*P* = 0.0399) (See Supplementary Figure 1) for eEC and CAH after HR are significantly higher than LNG-IUS alone. There was no statistical difference in the regression (*P* = 0.4098) (See Supplementary Figure 2) and recurrence (*P* = 0.8561) (See Supplementary Figure 3) rate between these two methods (See Supplementary Table 1).

### Oral progestogens alone for eEC and CAH

Women with eEC treated with oral progestogens alone had a pooled regression, recurrence and live birth rate of 79.47% (95% [CI], 73.19–85.10), 27.34% (95% [CI], 18.19–37.56) and 32.28% (95% [CI], 22.87–42.48), respectively. A pooled regression, recurrence and live birth rate of 88.74% (95% [CI], 81.70–94.25), 9.20% (95% [CI], 3.91–16.43) and 28.74 % (95% [CI], 19.20–39.35), respectively, was achieved in patients with CAH who were treated with oral progestogens only. Compared to those with eEC, women with CAH achieved a statistically significant higher regression (*P* = 0.0417) and a relatively lower recurrence rate (*P* = 0.0044) but no difference in live birth rates (*P* = 0.7247) when treated with oral progestogens (See Supplementary Table 2).

## DISCUSSION

The efficacy and safety of oral progestogens as fertility-sparing therapy in patients with eEC and CAH has been reported in many small studies, the first report was in 1961 by Kelley and Baker [63]. It functions by inhibition of the estrogen receptor, leading to a decrease in endometrial cell mitosis, promotion of apoptosis, and production of secretory endometrium.

The overall complete response rate for both eEC and CAH ranges from 62.5 to 89% [17, 48, 52, 53, 64]. In 2010, Serkanli and Ayhan [65] reviewed 231 cases and the overall response rate was 75.3% (*n* = 174), and another recent meta-analysis showed a response rate of 72% (95% CI 62–80%) [66]. It is reported that the regression rate for eEC and CAH is 57%–75% and 83%–94%, respectively [1, 22]. A pooled regression rate of 76.2% for eEC and 85.6% for CAH was reported in a meta-analysis made by Gallos et al. [8]. In our study, we report a total pooled regression rate of 88.74% for CAH and 79.47% for eEC, which is similar to previously published studies and found that the success rate is lower in women with eEC than those with CAH, the same as reported in Hara’ article [23].

The recurrence rates for eEC and CAH in Gallos’ meta-analysis was 40.6% and 26%, respectively [8]. A lower total pooled relapse rate of 27.34% for eEC and 9.20% for CAH was obtained from our meta-analysis. Reasons may be that we only exact the G1 EC other than all grades in their report, which affects the recurrence rate of the disease. While successful regression is important, the live birth rate is crucial to justifying the efficacy of fertility-sparing therapies. We found a pooled live birth rate of 33.38%, comparable to the rate of 28% in Gallos et al. meta-analysis [8].

The LNG-IUS acts on the progesterone receptors in the endometrium directly, thus the concentration of progesterone has been found to be much higher in the endometrial mucosa [67]. Studies focusing on the efficacy and safety of the LNG-IUS are also insufficient, and many use it only in conjunction with oral progestogens, although several recent studies have shown satisfying results [5, 29, 67–69]. Furthermore, some studies suggest that the LNG-IUS is superior to oral progestogens, Gallos et al. [5] reported a systematic review that LNG-IUS achieved a higher pooled regression rate than oral progestogens for CAH (90 vs 69%, *P* = 0.03). A higher regression rate (53/53, 95% CI 0.93–1.0) was also seen in a randomized, multicenter study carried out by Orbo et al. [67]. Similar result also reached in our study, a higher pooled regression rate of 94.24% vs 77.20% (*P* = 0.0010) for LNG-IUS compared to oral progestogens. This is similar to a study by Kim et al. [29] in which an 87.5% (14/16) complete response rate was obtained. However Baker et al. [10] found that treatment with oral or intrauterine progestogens is similarly effective, similar conclusion is reported in several studies [5, 32].

The recurrence rate of LNG-IUS in our study is 3.90%, which is significantly lower than oral progestogens alone (*P* = 0.0001). The live birth rate is 18.09% without significant difference with oral progestogens (*P* = 0.1242)(See Supplementary Table 3) suggesting LNG-IUS may still be a useful treatment option, especially since it has less systemic side-effects such as weight gain and irregular vaginal bleeding [70].

Hysteroscopic resection (HR) as a fertility-sparing treatment for eEC and CAH is less frequently used. Most articles report on HR followed by oral progestogens or GnRH agonists. A recent updated meta-analysis reported the regression rates of hormones only, surgery only, and hormones and surgery combined as 49.6% (111/224), 75% (3/4), and 100% (3/3), respectively [65]. The superiority of the combination is evident. A similar regression rate to our 97.25% can be seen with other published studies [6, 32, 71]. In comparison with oral progestogens, a statistically significant higher response rate (98.06% vs 77.20%) was achieved in our study. Our recurrence rate was 4.79%, similar to the 7% rate in Laurelli’s study [71], but lower than 30% for CAH and 27.3% for EC in Shan et al. [46]. The live birth rate reported in previous studies varies from 25% to 66.6% [6, 46], similar to our finding 52.57%. The potential superiority of the HR followed by hormonal therapy compared with oral progestogen use alone is evident.

HR consisted of three steps: excluding the lesion areas, the nearly endometrium and the myometrium under the lesion [6, 32, 71]. This operation could lead to accuracy diagnosis of the pathology and myometrial invasion. HR also helps improve the efficacy of progestogen due to less tumor burden. As a result, a higher regression rate and a shorter time between diagnosis and regression [72]. Some complication of HR such as endometrial destruction, intrauterine adhesion, which can affect the reproductive outcomes of young patients desire to preserve their fertility as mentioned before [73]. It is reported that in the study of Marzi et al. [32], a study aimed to evaluate the rate of intrauterine adhesions of HR as fertility-sparing therapy and found no intrauterine adhesions at the follow-up diagnostic hysteroscopy even in patients accepted more than once HR. Meanwhile, diagnostic hysteroscopy can found the uterine synechia caused by serial dilation and curettage (D&C) at follow-up and operate hysteroscopic adhesiolysis [22]. A recent prospective study focused on HR and progestin therapy reached a live birth rate of 50% in all treated patients and 86.6% for women who tried to conceive with no complications [72]. Another problem may be the dissemination of cancerous cells into the peritoneal cavity during hysteroscopy, it is reported that it didn't risk recurrence rate and the long-term prognosis is unclear [16, 74, 75]. Far from satisfactory, the low fertility rates can be explained by the fact that many women diagnosed with eEC or CAH are overweight, obese, anovulation or have polycystic ovarian syndrome all of which significantly impact the pregnancy rate [1, 22, 24, 76]. It is reported that a higher pregnancy and live birth rate in use of Assisted Reproductive Technology(ART) than spontaneous conception in young women with EC [14, 40, 72]. Patients who reached regression with fertility sparing therapy, immediately conceive should be suggested especially under ART.

The standard therapy for women with eEC and CAH still TH and BSO, so definite surgery is strongly suggested once a persisted or progression disease was found or after finishing delivery. We extracted the patients who undergone definite surgery and the final pathology. A total of 381 patients accepted standard therapy of which 37 patients (9.7%) showed no residual lesion, while persistent disease was seen in 219 women (57.5%). Unfortunately, ninety patients (23.6%) were diagnosed with progression when TH and BSO was operated and 6.5% (25) women with concurrent ovarian cancer. A recent study aimed at the EC developing risk between young women with CAH accepted fertility-sparing therapy and primary hysterectomy, finding that fertility-sparing therapy delays the occurrence of EC without increasing its risk [73]. The incidence of concurrent ovarian cancer was 5% as reported [77], a similar rate in our study. To exclude ovarian cancer before fertility sparing therapy, serum CA125 and diagnostic laparoscopy was used [62, 72, 78]. About 5% to 10% young patients with EC may be a candidate for Lynch syndrome [15, 16, 61, 72], so that it is essential to evaluate it before conservative treatment. According to our search, only seven studies performed.

The limitations of this study take into account the publications on which they were based. Publication bias exists due to possible overestimation of reported success rates. Insufficient studies on HR and LNG-IUS is another defect. Many of these reports are retrospective and include small sample sizes with limited follow up. Although fertility-sparing therapy does appear to be effective for women with eEC and CAH, with satisfactory regression and live birth rates, the recurrence rate is still concerning. Close, long-term surveillance should be performed.

In conclusion, hysteroscopic resection followed by progestin as fertility-sparing therapy may be a more effective option for women with CAH or EC. Further studies are needed to evaluate HR with or without hormonal therapy or a LNG_IUS, as well as the combination of LNG-IUS and oral .

## MATERIALS AND METHODS

### Identification of literature

The population of interest is young women aged 44 years or younger with early stage (International Federation of Gynecology and Obstetrics stage I) Grade 1 EC or CAH, who underwent fertility-sparing therapy including MA and MPA, the LNG-IUS, and hysteroscopic resection(HR) followed by progestogens. The outcome of interest was incidence of disease regression, recurrence and live birth . We searched Medline, the Cochrane Library and Embase (from January 1950 to December 2016) with the following Medical Subject Headings (MeSH) and text words: i) Words with sense of EC: “cancer” or “carcinoma” or “adenocarcinoma” or “malignant” or “neoplasm” AND “endometrial” or “endometrium” or “corpus uteri”. ii) Words with a similar meaning to CAH: “precancer” or “precursor” or “premalignant” or “precancerous” or “atypical hyperplasia” AND “endometrial” or “endometrium” or “corpus uteri”. iii) Therapies associated with fertility-sparing treatment: “fertility sparing” or “fertility preserve” or “fertility preserving” or “fertility preservation” or “conservative” or “hysteroscopic resection” or “GnRH analogue” or “hormone therapy” or “progesterone” or “progestin” or “levonorgestrel-releasing intrauterine system”. The following words were used to generate a subset of citations “humans and female” “age between 19–44 years old” and “language in Chinese and English”.

### Study selection and data extraction

Inclusion criteria were defined as: 1) Women between 19 and 44 years old;2) patients who desire fertility; 3) diagnosis of stage I grade I EC or CAH ; 4) treatment with fertility-sparing therapy and 5) articles written in English and Chinese. Exclusion criteria consisted of: 1) Women aged 45 or older; 2) those who underwent conservative treatment due to high perioperative risks; 3) patients with greater than stage IA disease(invading deep myometrium or distant metastasis) or pathology other than endometrioid adenocarcinoma; 4) patients with simple hyperplasia or complex hyperplasia without atypia; 5) case reports and small studies with fewer than five patients; 6) articles written in languages other than English and Chinese; 7) data was unable to be extracted.

Studies were selected in two stages. Firstly, the titles and abstracts of the articles searched from Medline, the Cochrane Library and Embase were independently assessed by two reviewers. Secondly, full articles meeting the inclusion criteria according to the first step were obtained and evaluated. Any disagreements about inclusions were arbitrated by a third reviewer. Two reviewers performed the quality assessment and adhered to the Methodological Index for Non- Randomized Studies (MINORS), a widely accepted tool used to assess the quality of the included studies [79].

Disease regression was defined as eEC or CAH returned to normal endometrium or hyplasia without atypical during follow-up. Disease recurrence was defined as eEC or CAH reoccurred during follow-up in patients initially showed disease regression. Live births was the birth of healthy infants during the follow-up period, and its rate was calculated as the number of women who gave birth of healthy infants divided by the number of women accepted fertility-sparing therapy and wanted to pregnant immediately. We defined appropriate follow-up time to be at least 5 years.

### Statistical analysis

After data extraction, regression, recurrence and live birth rates were calculated separately. Both a fixed and random effects model was used to obtain pooled rates [80]. Heterogeneity of the effects was statistically analyzed using the *Q* test and *I*^2^ test [81]. The forest plots were used to demonstrate the meta-analysis directly [81]. The regression, recurrence and live birth rates between the two interventions (HR followed by progestogens and oral progestogens, HR followed by progestogens and LNG-IUS; oral progestogens and LNG-IUS) were compared with the aid of meta-regression. Packages of “meta” and “meta for” written in R were used for calculations [82].

## SUPPLEMENTARY MATERIALS FIGURES AND TABLES




