# Supplementary material for: Comparison among fertility-sparing therapies for well differentiated early-stage endometrial carcinoma and complex atypical hyperplasia
Source: Oncotarget. 2017 May 3;8(34):57642–53. doi: 10.18632/oncotarget.17588 (PMC5593673; doi:10.18632/oncotarget.17588)
Supplement: Supplementary file 1 [file oncotarget-08-57642-s001.pdf]

Comparison among fertility-sparing therapies for well differentiated early-stage endometrial carcinoma and complex atypical hyperplasia

Supplementary Materials

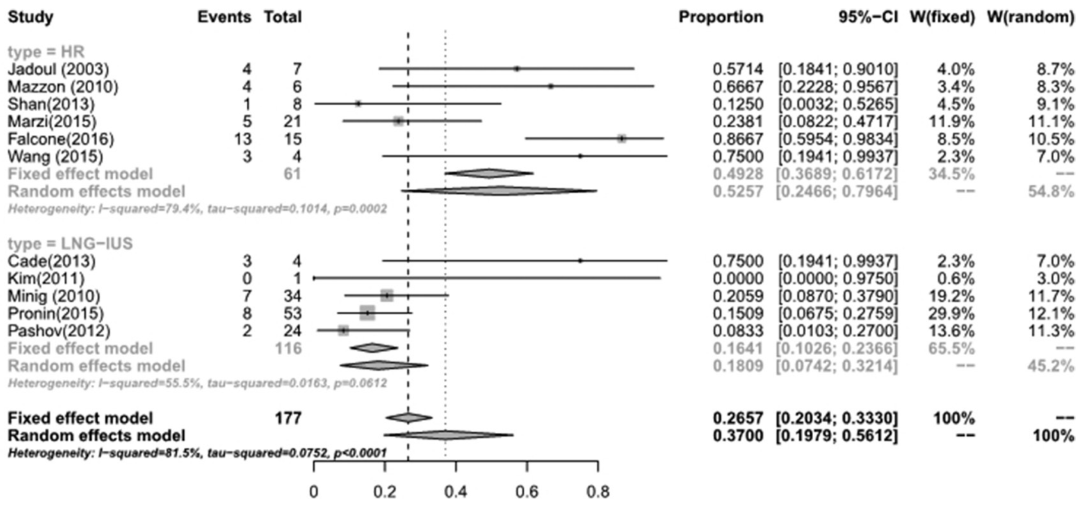

Supplementary Figure 1: Live birth rate between hysterscopic resection (HR) and LNG-IUS.

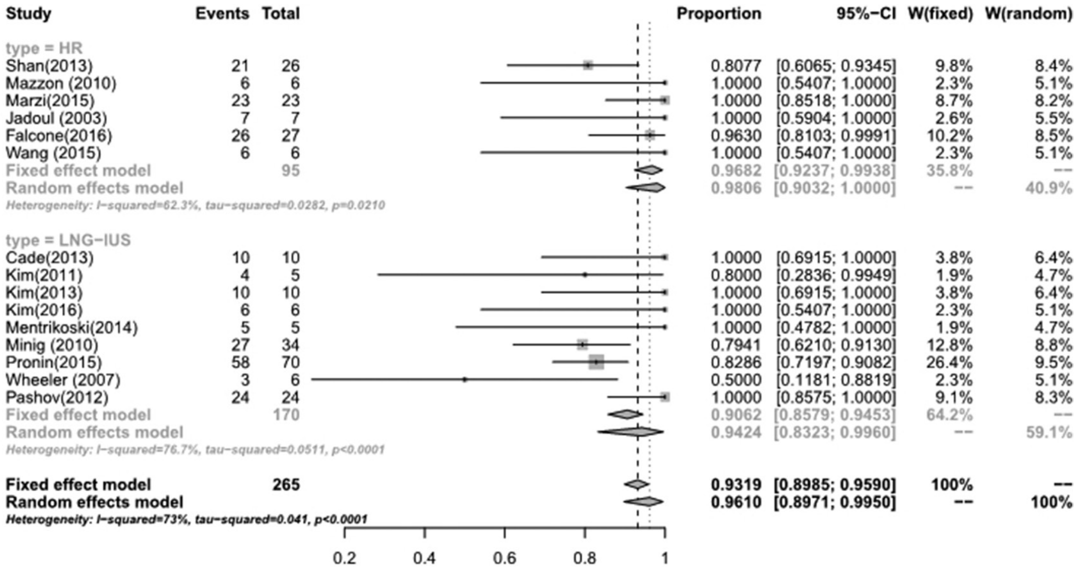

Supplementary Figure 2: Regression rate between hysterscopic resection (HR) and LNG-IUS.

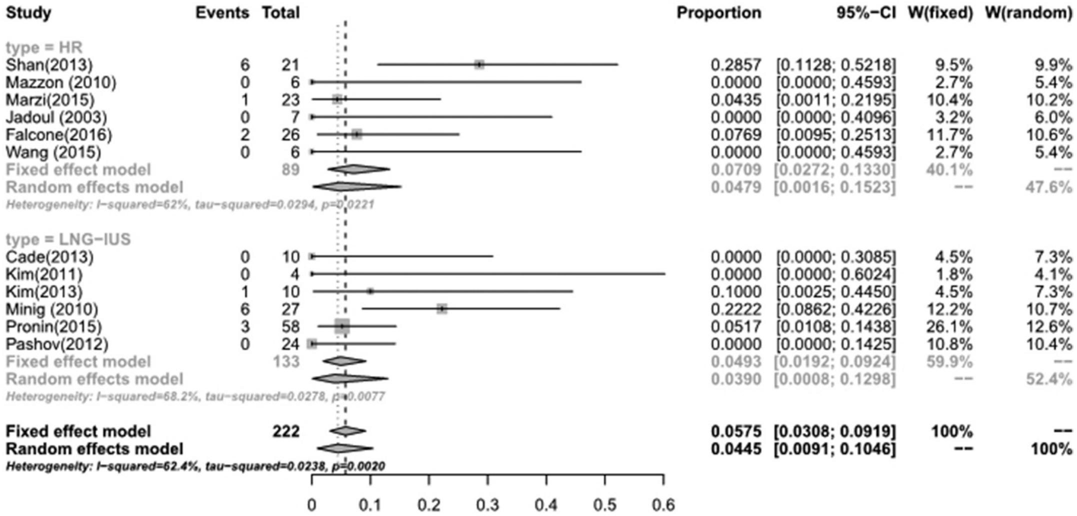

Supplementary Figure 3: Recurrence rate between hysterscopic resection (HR) and LNG-IUS.

Supplementary Table 1: Regression, recurrence and live birth rate of HR and levonorgestrel-releasing intrauterine system (LNG-IUS)

	Number of studies	Test for Heterogeneity (I^2)	Test for Heterogeneity: (*p* value of *Q* test)	Fixed effect model (effect size, 95% CI)	Random effects model (effect size, 95% CI)	*P* value
Regression rate						**0.4098**
In HR	6	62.3%	0.0210	0.9682 [0.9237; 0.9938]	0.9806 [0.9032;1.0000]	
In LNG-IUS	9	76.7%	< 0.0001	0.9062 [0.8579; 0.9453]	0.9424 [0.8323;0.9960]	
Recurrence rate						**0.8561**
In HR	6	62%	0.0221	0.0709 [0.0272; 0.1330]	0.0479 [0.0016;0.1523]	
In LNG-IUS	6	68.2%	0.0077	0.0493 [0.0192; 0.0924]	0.0390 [0.0008;0.1298]	
Live birth rate						**0.0399**
In HR	6	79.4%	0.0002	0.4928 [0.3689; 0.6172]	0.5257 [0.2466;0.7964]	
In LNG-IUS	5	55.5%	0.0612	0.1641 [0.1026; 0.2366]	0.1809 [0.0742;0.3214]	

Supplementary Table 2: Regression, recurrence and live birth rate of oral progestogens (P) for eEC and CAH

	Number of studies	Test for Heterogeneity (I^2)	Test for Heterogeneity: (*p* value of *Q* test)	Fixed effect model (effect size, 95% CI)	Random effects model (effect size, 95% CI)	*P* value
Regression rate						**0.0417**
For CAH	24	68.1%	< 0.0001	0.8916 [0.8557; 0.9229]	0.8874 [0.8170;0.9425]	
For eEC	40	71.1%	< 0.0001	0.7841 [0.7536; 0.8130]	0.7947 [0.7319; 0.8510]	
Recurrence rate						**0.0044**
For CAH	20	64%	< 0.0001	0.0883 [0.0562; 0.1268]	0.0920 [0.0391; 0.1643]	
For eEC	35	81.1%	< 0.0001	0.2764 [0.2386; 0.3157]	0.2734 [0.1819; 0.3756]	
Live birth rate						**0.7247**
For CAH	15	50.8%	0.0125	0.2739 [0.2093; 0.3436]	0.2874 [0.1920; 0.3935]	
For eEC	32	76.6%	< 0.0001	0.3520 [0.3076; 0.3978]	0.3228 [0.2287; 0.4248]	

Supplementary Table 3: Regression, recurrence and live birth rate of oral progestogens (P) and levonorgestrel-releasing intrauterine system (LNG-IUS)

	Number of studies	Test for Heterogeneity (I^2)	Test for Heterogeneity: (*p* value of *Q* test)	Fixed effect model (effect size, 95% CI)	Random effects model (effect size, 95% CI)	*P* value
Regression rate						**0.0010**
In LNG-IUS	9	76.7%	< 0.0001	0.9062 [0.8579; 0.9453]	0.9424 [0.8323;0.9960]	
In P	45	62.7%	< 0.0001	0.7712 [0.7454; 0.7960]	0.7720 [0.7258; 0.8151]	
Recurrence rate						**0.0001**
In LNG-IUS	6	68.2%	0.0077	0.0493 [0.0192; 0.0924]	0.0390 [0.0008; 0.1298]	
In P	39	76.5%	<0.0001	0.3029 [0.2708; 0.3359]	0.3217 [0.2506; 0.3971]	
Live birth rate						**0.1242**
In LNG-IUS	5	55.5%	0.0612	0.1641 [0.1026; 0.2366]	0.1809 [0.0742; 0.3214]	
In P	37	67.8%	<0.0001	0.3476 [0.3107; 0.3855]	0.3368 [0.2687; 0.4084]	

Supplementary Table 4: Characteristics of the studies. See Supplementary_Table_4
