# Supplementary material for: Comparison among fertility-sparing therapies for well differentiated early-stage endometrial carcinoma and complex atypical hyperplasia
Source: Oncotarget. 2017 May 3;8(34):57642–53. doi: 10.18632/oncotarget.17588 (PMC5593673; doi:10.18632/oncotarget.17588)
Supplement: Supplementary file 2 [file oncotarget-08-57642-s002.doc]

| Characteristics of the studies | | |  |  |  |
| --- | --- | --- | --- | --- | --- |
| Study | Total | Pathology | Imaging | Treatment | Following time |
| Baek (2016)^10^ | 31 | G1 EC（n=13）CAH(n=18) | MRI | MA 80-160 mg/d（n=25）MPA 40–120 mg/d（n=6） | 11.5m(3-29) |
| Bokhman (1985)^11^ | 19 | EC G1(n=11) G2(n=8) | Not mention | Hydroxyprogesterone 500mg/d | 3-9y |
| CADE (2013)^12^ | 10 | G1 EC | MRI | Oral progestogen 200 mg bid and/or intra-uterine progestogen | 89 m(62–142) |
| CAO (2013)^13^ | 51 | EC(n=38) CAH(n=13) | TUS MRI | MPA 250mg bid,500mg qd,MA 160mg bid/tid | Not mention |
| Chen（2016）^14^ | 53 | G1 EC(n=37) CAH(n=16) | TUS MRI | MPA 250–500 mg/d(n=32) MA 160–480 mg/d (n=16) + GnRH-a(n=9) LNG-IUS(n=2) | 54 m(4–148) |
| Dursun (2012)^15^ | 43 | G1 (n=34) G2(n=8) G3(n=1) | Not mention | MA (n=29) MPA (n=14) combined with IUD(n=7) | 3-29m |
| Duska (2001)^16^ | 12 | G1 EC | Not mention | Progestins at various dose | 82(6-358) |
| Eftekhar (2009)^17^ | 21 | G1 EC | US CT MRI | MA160 mg/d or MA 320mg/d+aspirin 80 mg/d | 48 m (18-84m) |
| Elizur(2007)^18^ | 8 | G1 EC | MRI | MA 160mg/d(n=6) MPA 200mg/d(n=1) 600mg/d（n=1） | 18 to 44 m |
| Fujiwara(2012)^19^ | 59 | EC(n=44) Ib-IIa(n=15) | MRI | MPA 400-600 mg/d | 66 m(11-251) |
| Gotlieb (2003)^20^ | 13 | G1(n=11)G2(n=1)G3(n=1) | MRI | MA 160mg/d(n=8)MPA200-600mg/d(n=2)NET5mg/d(n=1) | 82 m(6–358) |
| Hahn(2009)^21^ | 35 | G1(n=31)G2(n=4) | MRI | MPA 250-1500 mg/d(n=20) MA 160 mg/d(n=8) both (n=7) | 39 m (5-108 m) |
| Han (2009)^22^ | 10 | G1(n=5) G2(n=2)CAH(n=3) | TVUS MRI | MA (n=7) 80-160 mg/d MPA（n=3）500-1000 mg/d | 21m(9-51 )after delivery |
| Hara（2015）^23^ | 27 | EC(n=16) CAH(n=11) | MRI | MPA 400-600mg/d | 39.2 m (3.4–153.8) |
| Jadoul (2003)^24^ | 7 | EC G1(n=5) CAH(n=2) | Not mention | Endometrial resection and GnRH agonists | 26-40m |
| Kaku (2001)^25^ | 30 | G1(n=10)G2(n=2)CAH(n=18) | US CT MRI | EC:MPA 200–800 mg/d; ACH:MPA 100–800 mg/d | EC 24m(13-90)  CAH 45m(10-133 ) |
| Kataoka(2013)^26^ | 10 | EC(n=7) CAH(n=3) | TVUS | 400-600mg/d MPA 12w | at least 6 m |
| Kim (1997)^27^ | 7 | G1 EC | Not mention | MA 160mg/d | 7-46 m |
| Kim（2013）^28^ | 16 | G1EC | TVS MRI CT | LNG-IUS + MPA (500 mg/d). | 31.1±11.8 m (16-50) |
| Kim(2016)^29^ | 6 | CAH (n=6) | Not mention | LNG-IUS | 12m |
| KOSKAS(2012)^30^ | 22 | EC(n=8) CAH(n=14) | TUS MRI | NA, 17-hydroxyprogesterone derivate (MPA, MA or CA) and lynestrenol | 39 m(14-86) |
| Mao (2010)3^1^ | 6 | G1 EC | CT MRI TVUS | MPA 250-500 mg/d (n=4)MA 160 mg/d(n=2) | 32-77m |
| Marzi（2015）^32^ | 23 | EC(n=3) CAH(n=20) | CA125 MRI TUS | Hysteroscopic resection and MPA 20 mg/d (n=3) MA 160 mg/d (n=18) Levonorgestrel IUD (n=2) | 25m (8–37) |
| Mazzon (2010)^6^ | 6 | G1 EC | MRI TVUS | hysteroscopic resection followed by MA 160 mg/d | 50.5 m(21–82) |
| Mentrikoski(2014)^33^ | 13 | EC(n=10) CAH(n=3) | Not mention | MA IUD | 12 m(6-59) |
| Minaguchi (2007)^34^ | 31 | G1 EC(n=19) CAH(n=12) | CT MRI TVUS | MPA 2.5–600 mg/d (mostly 400-600 mg/d) | 40.7m(2-109 ) |
| Minig (2010)^35^ | 34 | G1 EC(n=14) CAH(n=20) | US MRI | LNG-IUS(20ug/d for 12m) and GnRHa(3.75mg) | 29 (4–102)m |
| Niwa (2005)^36^ | 12 | G1 EC | US MRI | Progesterone-releasing IUD (65ug/d) | 24-138m |
| Nomura(2016)^37^ | 18 | APA(n=14) EC(n=4) | MRI | MPA 400 mg/d(n=10) 600 mg/d (n=5) 200 mg/d(n=3) | 77.6 m(22–142) |
| Ota (2005)^38^ | 12 | G1EC | CT MRI US | MPA 600 mg/d | 52.7m（13–154) |
| Park (2012)^39^ | 14 | G1EC | MRI | MPA 250-500 mg/d (n=10) or Provera 30 mg/ d(n=2) or MA (16-240 mg/d (n=2) | 47.3±29.7 |
| Park(2013)^40^ | 177 | G1 EC | TUS MRI CA125 | MPA 500 mg/d (30–1500 mg/d)(n=85) MA 160mg/d (40–320 mg/d)(n=56) | 87m(45–159) |
| Penner（2011）^41^ | 40 | G1EC(n=14) CAH(n=26) | Not mention | Progestin therapy | Not mention |
| Perri (2011)^42^ | 27 | G1EC | CT MRI US | MA 160-320 mg/d (n=21), NET 5 mg/d (n=1) hydroxyprogesterone 2-3 g/w (n=2), and MPA 100-600 mg/d (n=3) | 57.4 m (7.8-412 m |
| Pronin（2015）^43^ | 70 | EC(n=32) CAH(n=38) | TUS MRI CA125 | CAH GnRHa EC GnRHa+IUD | 17m(1-45) |
| Randall (1997)^44^ | 33 | EC(n=14) CAH(n=19) | Not mention | MPA 10-30 mg/d or MA 40-160 mg/d (n=29),ovulation induction (n=2), Bromocriptine(n=1), oral contraceptive (n=1) for 3-12M | 69m(25–113) |
| Ricciardi(2012)^45^ | 15 | EC(n=1) CAH(n=14) | MRI US CA-125 | MA (80-160 mg/d) MPA(500-1000 mg/d) 12 w | Not mention |
| Shan（2013）^46^ | 26 | EC(n=14) CAH (n=12) | CA125 MRI TVUS | Thorough curettage with hysteroscopy followed by MA 160 mg/day 12w | 32 m(15–66) |
| Shan(2014)^47^ | 16 | CAH | TVUS | MA 160mg/d (n=8) MA 160mg/d +MET 0.5g/d(n=8) | 12-16m |
| Shirali(2012)^48^ | 16 | G1 EC | US CA125 | MA 160mg/d | Not mention |
| Shobeiri(2013)^49^ | 8 | G1EC | TUS CT MRI CA125 | MA 320mg 3m | 34.5 m( 11-72) |
| Simpson（2014）^50^ | 44 | G1EC(n=25) CAH(n=19) | Not mention | MPA≥100 mg/d MA≥80 mg/d(n=11) MPA 100 mg/d or MA 80 mg/d(n=31) | 39m(5–128) |
| Ushijima (2007）^51^ | 39 | G1EC(n=22) CAH(n=17) | MRI | MPA 600 mg/d with low-dose (80mg) aspirin | 76.5m(21–118) |
| Wang（2006）^52^ | 6 | EC | TUS CA125 | MA 160mg/d | 12-78m |
| Wang (2002)^53^ | 9 | G1 EC | US MRI | MA 160 mg/d and tamoxifen 30 mg/d for 6 m | 69 m(25-113) |
| Wheeler (2007)^54^ | 31 | EC（n=21）CAH(n=10) | Not mention | Oral progestogens or progesterone-releasing IUD | 48.8m(14–132) |
| Yahata (2005)^55^ | 8 | EC | US MRI | MPA 1800 mg/d for at least 3 m | 34.6m(7–114) |
| Yamazawa (2007)^56^ | 9 | G1 EC | CT MRI | MPA 400 mg/d | 82m(6–358) |
| Yang (2005）^57^ | 6 | G1 EC | CT MRI US | MA 160 mg/d | 39m(5–108) |
| Yu (2009)^58^ | 24 | EC (n=8） ACH(n=17) | CT MRI US | MPA 250-500mg/d for EC (n=8) 100-500mg/d for CAH (n=14) | 51m (38–75) |
| Zhou(2015)^59^ | 32 | G1EC(n=19) CAH(n=13) | TUS MRI CA125 | MPA 250 mg/d or MA 160 mg/d. Metformin (0.25 g tid) | 32m(10-92) |
| Pashov(2012)^60^ | 24 | G1EC(n=11) CAH(n=13) | US | CAH:6m 3.75 mg GnRHa+6 m LNG-IUS;EC 8m 3.75 mg GnRHa+12 months LNG-IUS | CAH:2-6y EC:44.39±6.47m |
| Falcone(2016)^61^ | 28 | EC G1 G2 | TVS MRI CA125 CT | HR+MA(n=6) or LNG-IUS(n=22) | 92m(6-172) |
| Wang (2015)^62^ | 6 | EC G1 | TVS MRI | HR+MA | 48.5m(2-9y) |

G1EC:Grade 1 endometrial carcinoma

CAH:complex atypical hyperplasia

MRI:magnetic resonance imaging

US:ultrasonography

TVS:transvaginal ultrasonography

CT:computed tomography

CA125:serum cancer antigen 125

MA:megestrol acetate

MPA:medroxyprogesterone acetate

HR:hysteroscopic resection

LNG-IUS:levonorgestrel-releasing intrauterine system
